# Colorimetric Measurement of Triglycerides Cannot Provide an Accurate Measure of Stored Fat Content in *Drosophila*

**DOI:** 10.1371/journal.pone.0012353

**Published:** 2010-08-24

**Authors:** Bader Al-Anzi, Kai Zinn

**Affiliations:** Division of Biology, California Institute of Technology, Pasadena, California, United States of America; University of Texas MD Anderson Cancer Center, United States of America

## Abstract

*Drosophila melanogaster* has recently emerged as a useful model system in which to study the genetic basis of regulation of fat storage. One of the most frequently used methods for evaluating the levels of stored fat (triglycerides) in flies is a coupled colorimetric assay available as a kit from several manufacturers. This is an aqueous-based enzymatic assay that is normally used for measurement of mammalian serum triglycerides, which are present in soluble lipoprotein complexes. In this short communication, we show that coupled colorimetric assay kits cannot accurately measure stored triglycerides in *Drosophila*. First, they fail to give accurate readings when tested on insoluble triglyceride mixtures with compositions like that of stored fat, or on fat extracted from flies with organic solvents. This is probably due to an inability of the lipase used in the kits to efficiently cleave off the glycerol head group from fat molecules in insoluble samples. Second, the measured final products of the kits are quinoneimines, which absorb visible light in the same wavelength range as *Drosophila* eye pigments. Thus, when extracts from crushed flies are assayed, much of the measured signal is actually due to eye pigments. Finally, the lipoprotein lipases used in colorimetric assays also cleave non-fat glycerides. The glycerol backbones liberated from all classes of glycerides are measured through the remaining reactions in the assay. As a consequence, when these assay kits are used to evaluate tissue extracts, the observed signal actually represents the amount of free glycerols together with all types of glycerides. For these reasons, findings obtained through use of coupled colorimetric assays on *Drosophila* samples must be interpreted with caution. We also show here that using thin-layer chromatography to measure stored triglycerides in flies eliminates all of these problems.

## Introduction

Obesity is a serious health problem in developed societies, affecting more than 35% of the adult population. Efforts to identify mutations in humans that account for a major proportion of the obesity phenotype in the general population have not been successful. By contrast, remarkable progress has been made in identifying and characterizing genes mutated in rodent models of obesity [1]. These include leptin and the leptin receptor. However, it is likely that many genes involved in the control of body weight and fat deposition remain to be discovered. Forward genetic screens in genetically accessible model organisms such as *Drosophila* and *C. elegans* can help to identify new regulators of fat storage and metabolism. Some of the molecular pathways known to be involved in mammalian fat store regulation are also important in flies [2], suggesting that *Drosophila* may represent a useful genetic model for mammalian obesity.

One method that is commonly employed for evaluation of stored fat in *Drosophila* is a coupled colorimetric assay [3]–[11], versions of which are available as kits from several manufacturers [12]–[14]. The coupled colorimetric assay was developed to measure triglycerides in soluble lipoprotein complexes in mammalian serum. It relies on hydrolysis of triglycerides by a lipoprotein lipase to generate glycerol, which is then phosphorylated by glycerol kinase to make glycerol-1-phosphate. This is in turn oxidized by glycerol phosphate oxidase to produce hydrogen peroxide. The amount of hydrogen peroxide generated by glycerol phosphate oxidation, which is proportional to the amount of glycerol produced by lipase cleavage of the triglyceride, is then read out using a peroxidase reaction to generate products called quinoneimines that absorb light in the visible range, between 450 and 600 nm.

Fats are triglycerides composed of a glycerol backbone that is covalently linked to three fatty acids. Serum triglycerides are soluble as a result of their enclosure within lipoprotein vesicles. However, pure fats, such as those found in droplets of stored fat within cells, are essentially insoluble in aqueous media. We show here that insoluble fat samples cannot be measured accurately using coupled colorimetric assays, probably because insolubility decreases the susceptibility of the fatty acid-glycerol bonds to cleavage by the lipases in the assay kits. As a consequence, the kits cannot accurately measure stored fat in extracts from crushed flies. Furthermore, the kits actually measure quinoneimines, which absorb visible light in the same wavelength range as *Drosophila* eye pigments. Thus, when crushed fly extracts are assayed, much of the measured signal is actually due to these pigments. Finally, the lipoprotein lipases used in the colorimetric assays also cleave non-fat glycerides such as monoglycerides (glycerol attached to one fatty acid) and diglycerides (glycerol attached to two fatty acids) [15]–[18]. The glycerol backbones liberated from these molecules are what is measured through the remaining reactions in the assays. As a consequence, when older versions of these assay kits are used to evaluate tissue extracts, the observed signal actually represents the amount of free glycerols together with all types of glycerides. A newer kit available from Sigma includes a solution with no lipase that allows measurement of the fraction of the signal that is due to free glycerols.

The purpose of this short communication is to provide experimental data documenting these problems, in order to demonstrate the inability of coupled colorimetric assays to accurately measure stored fat in *Drosophila*. Some of these issues would apply to measurement of stored fat in any organism. However, to the best of our knowledge, the assay has only been used for this purpose in the *Drosophila* system. Our findings should thus provide guidance for future work on fat storage in flies.

In most of the papers that describe the analysis of genes and cells affecting fat storage in *Drosophila*, coupled colorimetric assays were used to provide a quantitative measure of triglyceride content, but the flies in question were also examined using other assays such as Nile Red staining of sections to visualize fat droplets [6], [7], [10], [11]. Thus, it is likely that the genes in question actually do alter fat content, even if the triglyceride measurements are not accurate. However, a recent Resource paper in Cell [5] described an RNAi screen of ∼10,500 genes to identify gene products involved in control of fat content. The sole assay used to evaluate fat in this screen was the coupled colorimetric assay, and glycerols were not measured separately from glycerides. This raises the concern that some of the 516 candidate genes identified in this paper are not genuine regulators of fat storage, and that other genes involved in fat storage may have been missed.

## Materials and Methods

For butter, lard, glycerol, purified fly fat, tri, di-, and monoglycerides, samples were prepared for the coupled colorimetric assay using the procedures described by Pospisilik et al. [5], in which 10 mg of a given substance was mixed with 1 ml of PBS with 0.1% Triton X-100 (PBT). The mixture was then sonicated (five 30 sec. pulses at maximum intensity), boiled for 5 min. to facilitate the formation of an even suspension, and 20 µl aliquots were tested using the StanbioTriglyceride Liquicolor kit or Sigma Triglyceride kit (TR0100), following the manufacturer's instructions. When the samples were prepared for the TLC assay [7], 10 mg of each material was mixed with 1 ml of 2∶1 chloroform/methanol. The TLC procedure was exactly as described in ref. [7].

**Figure 1:**
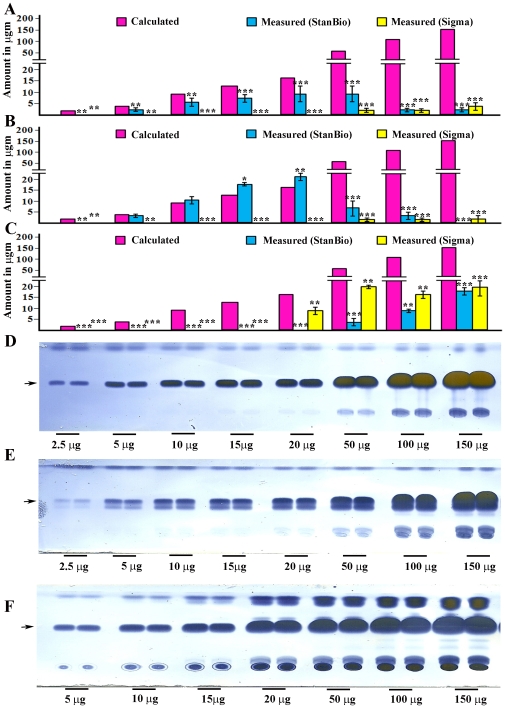
The coupled colorimetric assay is unsuitable for analysis of insoluble triglyceride mixtures. Lard (A), butter (B), and extracted fly fat (C) do not produce linear increases in quinoneimine absorption as more of each substance is assayed. Both the Stanbio Triglyceride Liquicolor Kit (blue bars) and the Sigma TR0100 kit (yellow bars) were used. The pink bars (Calculated) indicate the signal that would be observed if the samples could be measured accurately. Error bars are standard deviations of four different replicates for a given sample type. The butterfat and lard samples were sonicated and boiled as in ref. [5]. The extracted fly fat samples were resuspended without sonication and boiling, because sonicated and boiled samples generated even smaller signals (data not shown). Lard (D), butter (E), and extracted fly fat (F) all produce signals that increase with increasing amounts of either substance when assayed by the TLC method. The arrows indicate the location of the triglyceride band on the TLC assay. Asterisks denote T-test statistical significance: *; P<0.05–0,005, **; P<0.005–0.0005, ***; P<0.0005.

Partially purified *Drosophila* fat was obtained by crushing 200 flies in 2 mL of chloroform [19]. The homogenate was gently agitated for 1 hour at room temperature and spun down in an Eppendorff centrifuge at maximum speed for 15 min. 1.5 mL of the choloroform layer was taken and allowed to evaporate to dryness at room temperature. The resulting pellet was dissolved in 100 µL of 2∶1 chloroform/methanol, and the fat yield was estimated using the TLC assay with a known concentration of lard as a standard. For colorimetric measurements of the fly fat preparation, a measured amount of the solution was taken, and the chloroform/methanol solvent was allowed to evaporate, after which the samples were resuspended in PBT and assayed same as butter and lard samples. We assayed samples that were either resuspended only by vortexing (data shown in Fig. 1), or by sonication and boiling as described above (data not shown). Surprisingly, stronger colorimetric assay signals were observed when sonication and boiling were omitted.

Crushed fly samples (four replicates per experiment) were prepared using the same procedures [5]. Ten 5-day old males were crushed in 250 µL of PBT. The mixture was then sonicated and boiled as described above, then spun in an Eppendorff centrifuge at maximum speed for 15 min. 20 µL samples of supernatant were tested using the Stanbio or Sigma Triglyceride kits as described above. Samples were normalized to each other based on protein content, measured using the Bio-Rad BCA assay. For the TLC assay, the flies were crushed in 250 µL of 2∶1 chloroform/methanol mixture, spun down, and 2 µL of the supernatant was used for the TLC assay.

**Figure 2:**
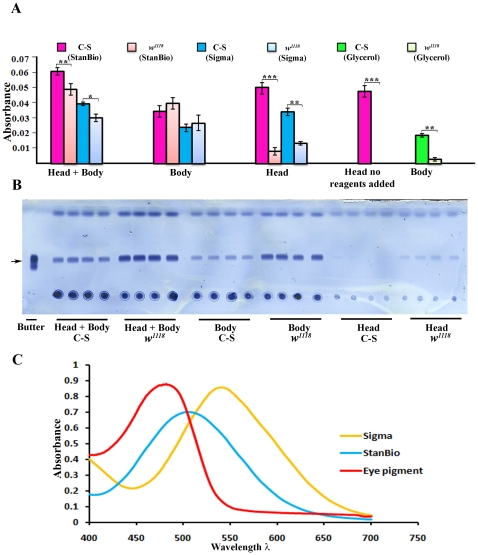
The coupled colorimetric triglyceride assay cannot accurately measure stored fat in adult *Drosophila*. Whole *w^1118^* (VDRC) flies contain more triglyceride than Canton S (C-S) flies, as shown by the stronger triglyceride bands (arrow) in (B) (head+body). Four replicates are shown. However, *w^1118^* produces weaker signals in the colorimetric assay than C-S (A, head+body bars, when either the StanBio or Sigma kits are used). The bars represent an average of the values from four replicates. The TLC assay shows that, as expected, heads have much less fat than bodies. *w^1118^* heads contain more fat than C-S heads (B, head). However, the colorimetric signal from C-S heads is much greater than that from *w^1118^* heads. The same signal strength is observed when C-S heads are assayed without any added enzymes, showing that this signal is due to absorption by eye pigment. *w^1118^* bodies produce a slightly stronger signal than C-S bodies, consistent with the TLC results; but the difference between C-S and *w^1118^* bodies in the colorimetric assay is not statistically significant. We also measured glycerols in C-S and *w^1118^* bodies, using the 'no lipase' reaction mix in the Sigma TR0100 kit. C-S bodies (bright green bars) contain much more glycerol than *w^1118^* bodies (light green bars). Error bars are standard deviations of four different replicates for a given sample type. Asterisks denote T-test statistical significance: *; P<0.05–0,005, **; P<0.005–0.0005, ***; P<0.0005. (C) Absorption spectra of quinoneimines produced by the StanBio and Sigma kits, and of fly eye pigment.

To examine the absorption spectra of the quinoneimines produced by the kit reactions, 50 µg of glycerol in PBT was mixed with 1 mL of either the Stanbio or Sigma Triglyceride kit solution, or with a blank made with either the Stanbio or Sigma reaction mix without glycerol. To generate the absorption profiles shown in Fig. 2, the values from the blank reaction were subtracted from those of the reaction with glycerol. To generate the eye pigment absorption spectrum, 50 µL of head homogenate of red-eyed (C-S) flies, or of white-eyed (*w^1118^*) flies was mixed with 850 µL PBT. The values obtained with the homogenate from white-eyed flies were subtracted from those obtained for the red-eyed flies. To facilitate comparison to the quinoneimine curves, the eye pigment absorption measurements were multiplied by 3 before graphing.

## Results

To evaluate the ability of coupled colorimetric assay kits to measure stored fat, we first examined whether butterfat or lard, used as examples of insoluble complex triglyceride mixtures whose composition resembles that of stored fat within cells, can be analyzed for triglyceride content using these assays. We also analyzed stored fat extracted from adult flies using chloroform [19]. We used assay kits from two manufacturers, StanBio and Sigma. The Sigma kit used was the newest version, TR0100, which allows separate measurement of glycerols and glycerides. In a previous paper [7], we reported that a non-aqueous thin-layer chromatography (TLC) assay, which allows direct visualization of triglyceride bands without the need for enzymatic conversion, can be used to evaluate stored fat in flies. Here we used TLC in parallel with the coupled colorimetric assays to analyze the butterfat, lard, and fly fat samples. As shown in Figs. 1A–C, the absorbance of the peroxidase product generated in the colorimetric assay does not increase linearly when increasing amounts of butterfat or lard are assayed. In fact, the signal decreases when amounts greater than 20 µg are used. The same amounts of butterfat or lard can be readily visualized using TLC (Figures 1D–E), and the intensity of the triglyceride band continues to increase with increasing butterfat or lard, up to the maximum weight assayed.

The partially purified fly fat sample also could not be accurately measured using the coupled colorimetric assay kits. It behaved differently from the butter and lard samples, in that signals were only observed when large amounts of fat (>20 µg) were used (Fig. 1C). On the TLC plate, the fly fat sample had a similar appearance to that of butterfat, although it contained more material (probably waxes) that did not migrate at all (Fig. 1F).

Next, we evaluated how the colorimetric triglyceride assay reading is affected by eye pigment absorption. We compared two fly lines, Canton S (red-eyed) and *w^1118^* (VDRC line, white-eyed), which differ in their fat content when analyzed using the TLC assay (Fig. 2B shows four replicate samples from each line). Quantization of the triglyceride bands using Photoshop indicates that *w^1118^* flies contain about 1.6-fold more triglyceride than Canton S flies, and this difference is statistically significant. However, when the colorimetric assay kits were used to assay the same fly lines, the apparent amount of triglyceride in the *w^1118^* flies was less than that in Canton S (Fig. 2A). In all experiments, the absorbance of the products of the kit reactions was measured at 540 nm, as specified in the instructions for the Sigma kit, even though the StanBio kit procedure calls for measurement at 500 nm. This was done because eye pigments absorb so strongly at 500 nm that they would completely obscure the signal (see Fig. 2C). The absorption curve for the quinoneimine generated by the StanBio kit is quite flat around 500–540 nm (Fig. 2C), so there is no significant loss in sensitivity when this kit is assessed at 540 nm rather than at 500 nm. Most of the fat in a fly is stored in the abdomen, so when heads are separated from bodies one would expect to find a much stronger triglyceride signal in the body than in the head. This is confirmed by the TLC assay (Fig. 2B), which also shows that there is a stronger triglyceride band for *w^1118^* bodies than for Canton S bodies. *w^1118^* heads have more triglyceride than Canton S heads. Examination of head vs. body fractions using the colorimetric triglyceride assay kits produces a completely different result. The amounts of triglyceride in the bodies from the two lines are similar. For both the StanBio and Sigma kits, there is a slight increase in the measured signal for *w^1118^* relative to Canton S, consistent with the TLC results, but this difference is not statistically significant (error bars overlap). However, a strong signal is observed for Canton S heads, greater than that seen for Canton S bodies (Fig. 2A). Almost no signal is seen for *w^1118^* heads. This result suggests that the majority of the signal in Canton S heads derives from absorption at 500 or 540 nm by eye pigments. We confirmed this by performing a 'blank assay' with the StanBio kit, taking the head extract through the assay steps without adding any enzymes. A signal of the same strength was observed under these conditions. This shows that fly eye pigments have strong absorption at the wavelengths used to measure the quinoneimine readout of the colorimetric triglyceride assays.

We compared the absorption spectra of the quinoneimines produced by the kits to that of eye pigments (Fig. 2C). These data show that eye pigment has an absorption maximum at ∼480 nm, but still absorbs strongly at 540 nm. We measured the output of the StanBio kit at 540 nm rather than 500 nm to reduce the background due to eye pigment absorption. Nevertheless, with either kit, the signal from the head is greater than that from the body, which is inconsistent with expectation and with the TLC results (Fig. 2B).

**Figure 3:**
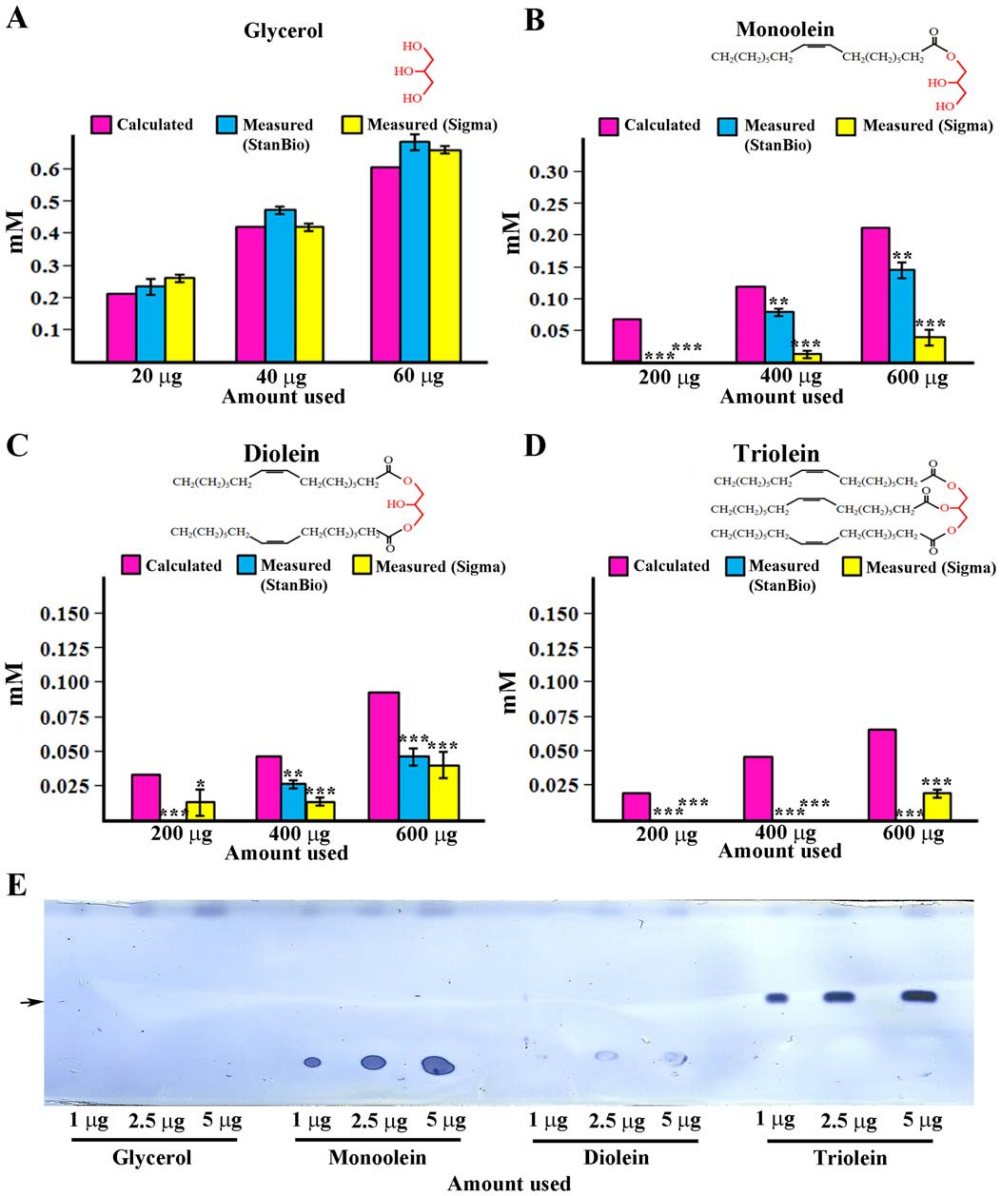
Coupled colorimetric assays cannot distinguish triglyceride from other glycerides. Glycerol (a soluble compound) can be accurately measured using either kit (A). Increasing amounts of monoolein (B) and diolein (C) produce increasing quinoneimine absorption with either kit. However, the values obtained are not in agreement with the known amounts (pink bars). Triolein (a pure triglyceride) produces no signal with the StanBio kit, and a small signal with the Sigma kit at the highest amount tested. (D). Much lower amounts of monoolein and triolein can be readily detected using the TLC assay (E) (arrow indicates the triglyceride band). Diolein bands do not stain well, and glycerol cannot be detected at all with the TLC assay. Cartoons of the glyceride structures are shown above the graphs in (A–D), with the glycerol backbone indicated in red. Error bars are standard deviations of four different replicates for a given sample type. Asterisks denote T-test statistical significance: *; P<0.05–0,005, **; P<0.005–0.0005, ***; P<0.0005.

The problem with eye pigment absorption can be addressed by separating bodies from heads before assaying fly tissue extracts. However, a third problem remains, which is that coupled colorimetric assays do not uniquely measure triglyceride. The colorimetric assay kits actually measure the amount of glycerol generated by the lipase reaction. Lipase cleavage would generate glycerol from nonfat mono- and diglycerides as well as from triglycerides. The colorimetric assays give accurate readings when used to measure triglycerides in mammalian serum lipoproteins because these other compounds are not present. They are, however, present in fly bodies. To evaluate whether the coupled colorimetric assay kits would also detect nonfat compounds in fly tissue extracts, we tested a monoglyceride (monoolein) and a diglyceride (diolein), and found that both produce signals (Fig. 3). Monoolein produces signals that correspond more closely to the calculated amount, possibly because it is more soluble and therefore more efficiently cleaved by the lipase. The triglyceride triolein cannot be assayed at all, probably due to insolubility. Free glycerol also produces a signal, and this corresponds exactly to the calculated amount since no cleavage is required. Glycerol-1-phosphate, and perhaps other glycerols, would also be expected to generate a signal. These compounds are all likely to be present in the bodies of flies, and could lead to a misinterpretation of the colorimetric assay results even if only bodies, or only white-eyed flies, are assayed. We examined glycerols in Canton S and *w^1118^* bodies using the 'no lipase' reaction in the Sigma TR0100 kit. Surprisingly, there is much more glycerol in the C-S sample than in the *w^1118^* sample (Fig. 2A). For Canton S, glycerol apparently accounts for about half of the total signal observed in bodies. This is further evidence that the results of the coupled colorimetric assays cannot be assumed to accurately report on stored fat content.

## Discussion

In this short communication, we show that failure to accurately measure stored (insoluble) fat is a general problem with using coupled colorimetric assays to evaluate fat content in *Drosophila*. These assays were originally devised (and are widely used) to measure triglyceride levels in human serum samples, and they are accurate for this purpose. The triglyceride in serum exists as soluble lipoprotein complexes, and the lipase used in colorimetric assay kits can efficiently cleave the glycerol head group from the fatty acid in these complexes.

When coupled colorimetric assays are used to evaluate suspensions of insoluble fat (butterfat, lard, or extracted fly fat), the lipase does generate some glycerol, but cleavage does not go to completion. Since cleaved glycerol is what is actually measured in the assay, this means that the measurement will be inaccurate. As the amount of insoluble fat in the sample increases, the divergence between the real amount and the measured amount increases, as shown in Fig. 1. The intensity of the triglyceride bands observed when crushed flies are examined by TLC (Fig. 2B) correspond approximately to 2.5–10 µg of insoluble fat. When these amounts of extracted fly fat were analyzed using the coupled colorimetric assay kits, no signal at all was observed (Fig. 1F).

A second problem with the assay is that absorption by eye pigment at the wavelengths (500 or 540 nm) used to measure the peroxidase reaction products that are generated by the colorimetric assays will cause fly lines with different eye colors to differ in their readings even if they do not have different levels of stored fat (Fig. 2). Eye pigment absorption accounts for ¼ to ½ of the measured signal when red-eyed flies are assayed. In the recent study by Pospisilik et al. [5], which describes high-throughput screening of ∼10,500 genes from the VDRC RNAi library using a coupled colorimetric assay kit, there is no indication that they performed any blank measurements to correct for eye pigment absorption. This should not have affected their initial screen for candidate fat regulatory genes, because they were comparing readings between flies in which RNAi construct expression is either induced (by heat shock) or not induced. These are the same fly lines, so they would have the same eye colors. However, they then examined which tissues are relevant to the effects on fat, by crossing different tissue-specific GAL4 drivers to the candidate RNAi lines. Since the various drivers and RNAi lines are all P element transgenics marked by w+ (mini-white), which confers variable eye colors depending on its insertion site, they can have different eye colors, and therefore would generate different eye pigment absorption signals when subjected to the colorimetric assays.

The third problem with using coupled colorimetric assays to measure triglyceride in tissue extracts is that they do not distinguish between triglycerides, diglycerides, and monoglycerides. The kits used in most published papers also do not distinguish between glycerides and glycerols (Fig. 3). This is not a problem in measurement of serum samples, since these other compounds do not contribute significantly to the measured readings. In *Drosophila*, however, lipid analysis (performed by Lipomics, Inc.) shows that there are substantial amounts of diglycerides (about ¼ of the amounts of triglycerides by weight) [20](B.A., unpublished results). The amounts of monoglycerides are unknown. We measured glycerols in two fly lines using the 'no lipase' reaction in the newest version of the Sigma kit. Surprisingly, the two lines had very different amounts of glycerols. For C-S flies, glycerol accounted for about half of the signal in the bodies (Fig. 2A). The measurement of glycerol separately from glycerides was apparently not performed in the Pospisilik et al. study [5], which used an older version of the Sigma kit.

We also note that the fact that the molecular weights of mono- and diglycerides and of glycerols are less than those of triglycerides means that these compounds will contribute more to the readings in the coupled colorimetric assays than would be suggested by their weight percentage in the tissue (that is, the molar amount of liberated glycerol, not the weight of the glyceride, is what is measured).

Most papers on analysis of specific genes and cells affecting fat content used Nile Red staining of fat droplets to confirm the results obtained with the coupled colorimetric assay, and it is likely that these droplets are composed primarily of triglycerides [6], [7], [10], [11]. The recent screen paper [5], however, used only a coupled colorimetric assay to evaluate fat content. Since they assayed >10,000 different genes, which may affect metabolism in many different ways, it is likely that some of these genes affect levels of other metabolites that would register in the assay. Thus, candidate genes that they report as affecting triglyceride levels may actually produce different readings in the colorimetric assay because they change the amounts of other glycerides or of glycerols.

### Implications for future studies of *Drosophila* fat storage

The main advantage of coupled colorimetric assay kits is that they are suitable for high throughput screening. In measuring fly fat, absolute amounts are often less important than the differences between lines in which specific genes have been mutated or manipulated. Since coupled colorimetric assays can give different readings when flies with different fat levels are assessed, they can provide a measure of the distinction between these flies even if the absolute amount of triglyceride is not measured accurately. However, bodies must be separated from heads before assay, to eliminate the eye pigment signal. Alternatively, if all the flies analyzed have the same eye color, a blank can be performed without enzymes, and this reading can be subtracted from all the other readings. This is less preferable, since it will reduce the sensitivity of the distinctions that can be made. Finally, it is important to use the newer Sigma kit and perform 'no lipase' reactions on all samples, since glycerols can account for a large percentage of the observed signal. Even if all of these precautions are taken, however, the assay results will still not distinguish triglycerides from other glycerides, and they will also not provide an accurate measurement of total glycerides due to the problem of insolubility. It is thus essential to use other methods such as TLC and Nile Red staining to confirm that any candidate genes identified using the coupled colorimetric assay actually affect triglyceride levels rather than altering the levels of other compounds that would register in the assay.

Our data indicate that TLC, which can be used in a semi-high-throughput manner to assess hundreds of lines [7], is a superior method for assessing fat content in Drosophila because: 1) it does not rely on enzymatic cleavage of the lipid and therefore is unaffected by lipid solubility (Fig. 1); 2) it is unaffected by eye color (Fig. 2); 3) it separates triglycerides from other glycerides (Fig. 3). It can also be quantitated using Photoshop analysis of images of TLC plates.
